# Acceptance of Telerheumatology by Rheumatologists and General Practitioners in Germany: Nationwide Cross-sectional Survey Study

**DOI:** 10.2196/23742

**Published:** 2021-03-29

**Authors:** Felix Muehlensiepen, Johannes Knitza, Wenke Marquardt, Jennifer Engler, Axel Hueber, Martin Welcker

**Affiliations:** 1 KV Consult- und Managementgesellschaft mbH Potsdam Germany; 2 Center for Health Services Research Brandenburg Medical School Theodor Fontane Rüdersdorf bei Berlin Germany; 3 Faculty of Health Sciences Brandenburg Brandenburg Medical School Theodor Fontane Neuruppin Germany; 4 Department of Internal Medicine 3 - Rheumatology and Immunology Friedrich-Alexander University Erlangen- Nürnberg and Universitätsklinikum Erlangen Erlangen Germany; 5 Institut für Allgemeinmedizin Goethe-Universität Frankfurt am Main Frankfurt am Main Germany; 6 Sektion Rheumatologie Sozialstiftung Bamberg Bamberg Germany; 7 Medizinisches Versorgungszentrum für Rheumatologie Dr M Welcker GmbH Planegg Germany

**Keywords:** telemedicine, eHealth, mHealth, rheumatology, primary care, health services research, COVID-19

## Abstract

**Background:**

The worldwide burden of musculoskeletal diseases is increasing. The number of newly registered rheumatologists has stagnated. Primary care, which takes up a key role in early detection of rheumatic disease, is working at full capacity. COVID-19 and its containment impede rheumatological treatment. Telemedicine in rheumatology (telerheumatology) could support rheumatologists and general practitioners.

**Objective:**

The goal of this study was to investigate acceptance and preferences related to the use of telerheumatology care among German rheumatologists and general practitioners.

**Methods:**

A nationwide, cross-sectional, self-completed, paper-based survey on telerheumatology care was conducted among outpatient rheumatologists and general practitioners during the pre-COVID-19 period.

**Results:**

A total of 73.3% (349/476) of survey participants rated their knowledge of telemedicine as unsatisfactory, poor, or very poor. The majority of survey participants (358/480, 74.6%) answered that they do not currently use telemedicine, although 62.3% (291/467) would like to. Barriers to the implementation of telemedicine include the purchase of technology equipment (182/292, 62.3%), administration (181/292, 62.0%), and poor reimbursement (156/292, 53.4%). A total of 69.6% (117/168) of the surveyed physicians reckoned that telemedicine could be used in rheumatology. Surveyed physicians would prefer to use telemedicine to communicate directly with other physicians (370/455, 81.3%) than to communicate with patients (213/455, 46.8%). Among treatment phases, 64.4% (291/452) of participants would choose to use telemedicine during *follow-up*. Half of the participants would choose *telecounseling* as a specific approach to improve rheumatology care (91/170, 53.5%).

**Conclusions:**

Before COVID-19 appeared, our results indicated generally low use but high acceptance of the implementation of telerheumatology among physicians. Participants indicated that the lack of a structural framework was a barrier to the effective implementation of telerheumatology. Training courses should be introduced to address the limited knowledge on the part of physicians in the use of telemedicine. More research into telerheumatology is required. This includes large-scale randomized controlled trials, economic analyses, and the exploration of user preferences.

## Introduction

The worldwide burden of musculoskeletal diseases is increasing [1]. Growing life expectancy, widespread overweight, and a frequent lack of exercise have caused a surge in musculoskeletal disorders. Besides individual health burden, these chronic diseases create a considerable financial burden for society overall, as patients often take sick leave and early retirement [2]. While increasingly effective treatments have been developed and implemented, the number of newly registered rheumatologists has stagnated [3], and the global need for rheumatologists cannot be met [4]. General practitioners are usually the first point of contact for patients and play an important role in the early detection of rheumatic and musculoskeletal diseases (RMDs). But primary care is also affected by a shortage of staff and, in view of demographic change, an increasingly demanding work burden [5,6]. A lack of physicians has led to diagnostic delays for various rheumatologic diseases [7] and a decrease in treatment efficacy [8].

In recent decades, information and communication technologies have entered health care. One field of application is telemedicine, which is defined as follows [9]:

Telemedicine is the practice of medicine over a distance, in which interventions, diagnoses, therapeutic decisions, and subsequent treatment recommendations are based on patient data, documents and other information transmitted through telecommunication systems.

The use of telemedicine in the provision of rheumatology care (telerheumatology) could ease constraints on health care access and the timeliness of care, bridge the workforce gap, and improve access to care for underserved communities [10,11]. The effectiveness of telerheumatology could be equal or higher than a standard face-to-face approach [12-16]. However, evidence is lacking, and further studies are needed to determine the best use of telerheumatology [16,17]. The current global COVID-19 pandemic has highlighted the need for telerheumatology and could promote the use of innovative solutions in clinical routines [18]. However, the effective implementation of telerheumatology requires acceptance by potential users.

The aim of this user-centered study was to assess the acceptance by physicians of the implementation of telerheumatology and to identify potential application areas in preparation for the development of telemedical approaches.

## Methods

This paper reports on findings from the analysis of data collected as part of a cross-sectional, self-completed, paper-based survey of German outpatient rheumatologists and general practitioners from September to November 2018; the survey investigated acceptance, opportunities, and obstacles to the implementation of telemedicine.

The inclusion criteria for participants were that they had to be (1) rheumatologists or general practitioners, (2) practicing in ambulatory health care, (3) based in Germany, and (4) active (ie, not retired and not in training).

Two health care researchers (FM and WM) and one experienced rheumatologist (MW) designed the questionnaire. It was pilot-tested on 5 rheumatologists and 5 general practitioners to gauge the need to refine wording and format, and to check whether predefined response options were exhaustive. Minor revisions were made accordingly.

The questionnaire comprised 25 questions and was divided into three mandatory sections:

1. Telemedicine: knowledge and use.
2. Telerheumatology.
3. Sociodemographic data.

Response categories were nominal or ordinal. The questionnaire also contained several open questions.

The *telemedicine*: *knowledge and use* section included questions about expertise and the use of telemedicine. Participants were asked to rate their knowledge of telemedicine on a 6-point Likert scale. Furthermore, inquiries were made into current telemedicine use, willingness to use telemedicine, and barriers to its use.

The *telerheumatology* section included questions about the perceived usefulness of telerheumatology. This section also included questions about preferences for particular uses of telerheumatology, such as patient groups, application areas, and specific tools.

Questions about sociodemographic and occupational characteristics included age, gender, medical specialty, clinical location, type of practice, and number of patients (quarterly).

The survey was sent to all rheumatologists (n=49) and general practitioners (n=1820) in the federal state of Brandenburg, Germany, and rheumatologists (n=39) and general practitioners (n=487) in a nationwide reference group. The contact details of potential participants in Brandenburg were provided by the Brandenburg Association of Statutory Health Insurance Physicians. The nationwide reference group consisted of physicians from RHADAR (RheumaDatenRhePort GbR) and cooperating rheumatologists and general practitioners. RHADAR is an association of more than 25 rheumatologists that share the aim of developing and improving digital technology. The association also collects and analyzes anonymized patient data to identify supply and demand for rheumatology services. In total, 88 rheumatologists and 2307 general practitioners were contacted by mail.

Statistical analyses were performed using SPSS Statistics for Windows, version 22.0 (IBM Corp). Descriptive statistics included quantities, percentages, median scores, and ranges for ordinal variables.

The study was conducted in compliance with current data protection regulations and the Helsinki Declaration. All study participants were informed about the research project. Data were anonymized before analysis. The ethics committee of the Theodor Fontane Medical School in Brandenburg stated that no written consent was necessary due to the noninterventional study design.

## Results

### Overview

From September to November 2018, a cross-sectional, self-completed, paper-based survey on telerheumatology was filled in by German outpatient rheumatologists and general practitioners. Of the 2395 questionnaires that were sent out, 497 (20.8%) were returned. Of the 497 responses, 12 (2.4%) were excluded from the analysis because fewer than half the questions were answered. The final response rate for rheumatologists was 55% (48/88) and for general practitioners it was 18.9% (437/2307).

### Sample Characteristics

Data for this study were obtained from 485 physicians (437/485, 90.1% general practitioners; 48/485, 9.9% rheumatologists) (see Table 1). About half of the respondents were between 50 and 60 years old (228/474, 48.1%). Slightly more than half of the participants were women (254/470, 54.0%). One-third of the surveyed physicians worked in a town (154/474, 32.5%), one-third worked in a provincial town (158/474, 33.3%), 17.9% (85/474) worked in a city, and 16.2% (77/474) worked in a rural area. Overall, 53.5% (252/471) of the physicians worked in a single-handed practice and 46.5% (219/471) worked in a group practice. Almost two-thirds of the surveyed physicians treated over 4000 patients per year.

**Table 1:** Participant demographics.

Demographic			Rheumatologists, n (%)^a^		General practitioners, n (%)^a^		*P* value		Total, n (%)^a^
**Age (years)**									
	Total	47 (100)		427 (100)		.22		474 (100)	
	<40	4 (9)		36 (8.4)				40 (8.4)	
	40-50	16 (34)		85 (19.9)				101 (21.3)	
	51-60	20 (43)		208 (48.7)				228 (48.1)	
	>60	7 (15)		98 (23.0)				105 (22.2)	
**Gender**									
	Total	46 (100)		424 (100)		.17		470 (100)	
	Female	19 (41)		235 (55.4)				254 (54.0)	
	Male	27 (59)		189 (44.6)				216 (46.0)	
**Clinical location**									
	Total	47 (100)		427 (100)		<.001		474 (100)	
	City	23 (49)		62 (14.5)				85 (17.9)	
	Town	10 (21)		144 (33.7)				154 (32.5)	
	Provincial town	14 (30)		144 (33.7)				158 (33.3)	
	Rural area	0 (0)		77 (18.0)				77 (16.2)	
**Type of practice**									
	Total	47 (100)		424 (100)		<.001		471 (100)	
	Single-handed	10 (21)		242 (57.1)				252 (53.5)	
	Group	37 (79)		182 (42.9)				219 (46.5)	
**No. of patients treated (quarterly)**									
	Total	45 (100)		411 (100)		.14		456 (100)	
	<500	4 (9)		15 (3.6)				19 (4.2)	
	500-1000	17 (38)		130 (31.6)				147 (32.2)	
	>1000	24 (53)		266 (64.7)				290 (63.6)	

^a^Percentages may not add up to 100% due to rounding.

### Telemedicine: Knowledge and Use

A total of 73.3% (349/476) of respondents rated their knowledge of telemedicine as 4 (unsatisfactory), 5 (poor), or 6 (very poor). The minority (127/476, 26.7%) rated their knowledge of telemedicine as 1 (very good), 2 (good), or 3 (satisfactory). The majority (358/480, 74.6%) did not currently use telemedicine, but 62.3% (291/467) answered that they would like to use it (see Table 2). A total of 89.3% (259/290) of surveyed physicians indicated that barriers prevented them from using telemedicine. The top three obstacles to the introduction of telemedicine according to respondents were the purchase of technology equipment (182/292, 62.3%), administration (181/292, 62.0%), and inadequate remuneration (156/292, 53.4%).

**Table 2:** Telemedicine: knowledge and use.

Question and responses			Rheumatologists, n (%)^a^		General practitioners, n (%)^a^		*P* value		Total, n (%)^a^
**How do you rate your own knowledge of telemedicine?**									
	Total	47 (100)		429 (100)		.14		476 (100)	
	1 (very good)	1 (2)		19 (4.4)				20 (4.2)	
	2 (good)	8 (17)		34 (7.9)				42 (8.8)	
	3 (satisfactory)	9 (19)		56 (13.1)				65 (13.7)	
	4 (unsatisfactory)	9 (19)		110 (25.6)				119 (25.0)	
	5 (poor)	16 (34)		138 (32.2)				154 (32.4)	
	6 (very poor)	4 (9)		72 (16.8)				76 (16.0)	
**Do you use telemedicine?**									
	Total	47 (100)		433 (100)		.15		480 (100)	
	Yes	16 (34)		106 (24.5)				122 (25.4)	
	No	31 (66)		327 (75.5)				358 (74.6)	
**Would you like to use telemedicine?**									
	Total	46 (100)		421 (100)		.45		467 (100)	
	Yes	31 (67)		260 (61.8)				291 (62.3)	
	No	15 (33)		161 (38.2)				176 (37.7)	
**Does anything prevent you from using telemedicine?**									
	Total	30 (100)		260 (100)		.26		290 (100)	
	Yes	25 (83)		234 (90.0)				259 (89.3)	
	No	5 (17)		26 (10.0)				31 (10.7)	
**What prevents you from using telemedicine? (multiple selections possible)**									
	Total	31 (100)		261 (100)				292 (100)	
	Purchase of technology equipment	16 (52)		166 (63.6)		.19		182 (62.3)	
	Administration	21 (68)		160 (61.3)		.49		181 (62.0)	
	Poor reimbursement	21 (68)		135 (51.7)		.09		156 (53.4)	
	Data security	15 (48)		120 (46.0)		.80		135 (46.2)	
	Lack of participation by colleagues	8 (26)		89 (34.1)		.35		97 (33.2)	
	Technical comprehension of patients	12 (39)		84 (32.3)		.47		96 (32.9)	
	Poor internet connection	5 (16)		52 (19.9)		.61		57 (19.5)	

^a^Percentages may not add up to 100% due to rounding or where multiple selections were possible.

### Implementation of Telerheumatology

A total of 69.6% (117/168) of participants considered telemedicine as usable in rheumatology (see Table 3).

When asked who should interact using telemedicine, 81.3% (370/455) responded *physician-physician*, 46.8% (213/455) responded *physician-patient*, and 25.7% (117/455) responded *physician-assistant* (multiple replies were possible). The preferred therapy phases for the use of telemedicine were *follow-up* (291/452, 64.4%), *initial contact* (153/452, 33.8%), and *screening* (133/452, 29.4%). Participants were asked to indicate specific digital tools that could support rheumatology care. The most frequently selected items were *telecounseling* (91/170, 53.5%), *telediagnostics* (76/170, 44.7%), and *video consultations* (67/170, 39.4%). These were followed by *online appointment assignments* (56/170, 32.9%), *e-learning* (55/170, 32.4%), *patient apps* (48/170, 28.2%), *digital screening* (36/170, 21.2%), *wearable devices* (20/170, 11.8%), *telesurgery* (7/170, 4.1%), and *other tools* (4/170, 2.4%).

**Table 3:** Implementation of telemedicine in rheumatology care.

Question and responses			Rheumatologists, n (%)^a^		General practitioners, n (%)^a^		*P* value		Total, n (%)^a^
**Is telemedicine usable in rheumatology?**									
	Total	43 (100)		125 (100)		.18		168 (100)	
	Yes	32 (74)		85 (68.0)				117 (69.6)	
	No	11 (26)		40 (32.0)				51 (30.4)	
**Which parties should establish communication via telemedicine? (multiple selections possible)**									
	Total	45 (100)		410 (100)				455 (100)	
	Physician-physician	36 (80)		334 (81.5)		.88		370 (81.3)	
	Physician-patient	28 (62)		185 (45.1)		.03		213 (46.8)	
	Physician-assistant	14 (31)		103 (25.1)		.38		117 (25.7)	
	Other participants and combinations	5 (11)		13 (3.2)		.009		18 (4.0)	
	No communication	3 (7)		54 (13.2)		.21		57 (12.6)	
**At which stages can telemedicine support rheumatology care? (multiple selections possible)**									
	Total	45 (100)		407 (100)				452 (100)	
	Screening	23 (51)		110 (27.0)		.001		133 (29.4)	
	Initial contact	11 (24)		142 (34.9)		.16		153 (33.8)	
	Follow-up	32 (71)		259 (63.6)		.32		291 (64.4)	
	Other stages	7 (16)		38 (9.4)		.19		45 (10.0)	
	At no stage	8 (18)		69 (17.0)		.90		77 (17.1)	
**Which tools could support rheumatologic care? (multiple selections possible)**									
	Total	44 (100)		126 (100)				170 (100)	
	Telecounseling	24 (55)		55 (43.7)		.21		79 (45.5)	
	Telediagnostics	18 (41)		58 (46.0)		.56		76 (44.7)	
	Video consultations	19 (43)		48 (38.1)		.55		67 (39.4)	
	Online appointment assignments	20 (45)		36 (28.6)		.04		56 (32.9)	
	e-Learning	15 (34)		40 (31.7)		.78		55 (32.4)	
	Patient apps	17 (39)		31 (24.6)		.08		48 (28.2)	
	Digital screening	15 (34)		21 (16.7)		.02		36 (21.2)	
	Wearable devices	9 (20)		11 (8.7)		.04		20 (11.8)	
	Telesurgery	3 (7)		4 (3.2)		.30		7 (4.1)	
	Other tools	2 (5)		2 (1.6)		.27		4 (2.4)	
	No tools	6 (14)		15 (11.9)		.76		21 (12.4)	

^a^Percentages do not add up to 100% where multiple selections were possible.

## Discussion

### Principal Findings

To the best of our knowledge, we have performed the largest nationwide survey in Germany on the use of telemedicine in adult rheumatology aimed at promoting and guiding the implementation of telerheumatology.

Rheumatologists and general practitioners generally consider the overall application of telerheumatology to be acceptable, and two-thirds of the respondents would like to implement telemedicine in their everyday practice. Rheumatologists expressed an even greater willingness to use telemedicine than general practitioners. Rheumatologists and general practitioners welcomed a wide variety of approaches to telemedicine. However, only a minority of physicians already used telemedicine at the time the survey was conducted. Barriers to its introduction, such as limited knowledge, administrative expenses, the purchase of technology equipment, and inadequate reimbursement, were clearly identified by specialists and generalists. The results provide information on how telemedicine can support rheumatology care from the physician’s perspective. Conservative and familiar communication formats, such as the exchange of information with colleagues, were preferred to the direct exchange of information with patients and assistants. This was demonstrated by the high approval of the item *telecounseling*. Although various telecounseling tools already exist, their development for rheumatology applications is not as advanced as in, for example, intensive care and cardiology. This is reflected in the relatively small number of respondents that were using telemedicine at the time of the survey.

### Limitations

The average age of our sample is similar to that of German physicians as a whole [19]. Women were slightly overrepresented compared to the average [19], which may also indicate that female physicians are more interested in telehealth. Due to the small number of rheumatologists, this survey mainly reports on the opinion of general practitioners. Although the survey was directed at physicians from all over Germany, it was primarily physicians from Brandenburg who participated due to the recruitment strategy. We assume a self-selection bias and a nonresponse bias, as the survey was probably mainly answered by physicians who are interested in telemedicine and rheumatology. To overcome this bias, we chose a paper-based survey. An online survey may have increased the response rate and reduced the effort for data management. However, we assumed an online survey would have influenced response behavior, in the sense of a positive bias toward users of telemedicine. To answer the questionnaire, knowledge in the field of telemedicine was required, as, for example, preferences for specific tools were asked about. Considering the limited knowledge of doctors in the field of telemedicine, response biases are, therefore, likely. Furthermore, we assume that due to rapid technical developments in the field, our predefined response categories were not exhaustive. Only ambulatory physicians were asked to participate in the survey, so the results do not provide any information on the acceptance by patients, hospital staff, or other professional groups other than outpatient physicians. Lastly, the survey was conducted in pre-COVID-19 times, so further research on the development of acceptance of telerheumatology use is highly needed.

### Comparison With Prior Work

This work adds to the growing body of telerheumatology knowledge [11-17] by providing detailed user preferences, needs, and barriers. Hence, we believe that the results of this study may help in the development of telemedicine solutions that can be integrated into the clinical routine of patients with rheumatic diseases.

In contrast to the results of a recent study that identified a negative attitude toward digitization in the health care system among physicians in Germany [20], our results indicated that physicians have a positive attitude toward telemedicine. An American Medical Association survey of 3500 physicians in the United States found that fewer than 10% of rheumatologists used telemedicine, which is significantly less than physicians from other medical fields, such as radiologists (43%) [21], and less than the percentage of rheumatologists that use telemedicine according to our study (34%). Although most respondents reckoned that telecounseling may support rheumatology care, telecounseling is not used or is rarely used. In a nationwide survey of digitization among German medical practices, only 1% of surveyed physicians responded that they use videoconferences to communicate with other physicians. The most frequent answers were *email* and *no digital communication at all* [22]. In the survey on digitization in German medical practices, security gaps in information technology (IT), the considerable cost and effort involved in introducing digital technologies, and an unfavorable cost-benefit ratio were identified as the main obstacles to digitization from the perspective of physicians [22]. Participants in our survey attached relatively little importance to security gaps in IT, with only 46.2% choosing *data security* as an issue that prevented them from using telemedicine. Televisual consultations with patients appear to have considerable potential in rheumatology care, and especially in initial consultations [12]. However, only a minority of participants in our survey favored the use of telemedicine in initial consultations. This finding confirms the results of a comparable study on veteran rheumatology care from the United States [23]. Furthermore, only one-third of those surveyed wished to use telemedicine in direct patient contacts at all, which contrasts starkly with overall telemedical developments in the health care system [16,17]. This is unfortunate, as previous research has shown that patients with rheumatoid arthritis consider telerheumatology to be a flexible solution that increases the independence of health authorities and raises personal knowledge [24]. Other studies indicate that health care resulting from televisits is as effective as that provided following in-person visits [25,26]. A qualitative study also reports that patients would be willing to accept electronic collection and sharing of patient-reported outcomes (PROs) between clinical encounters if it facilitated communication with health care providers and provided access to reliable information on RMDs [27]. However, a recent study has shown that rheumatologists are reluctant to study electronic PROs because it would lead to a massive increase in their workload [28].

Mobile apps promise to accelerate diagnostic investigations and improve monitoring and the outcomes of patients with RMDs [29]. The small number of rheumatologists that supported the use of apps to improve clinical routines (34%) contrasts with previous research from 2018 in which 49% said they were already using such apps [30]. One of the top reasons for the reluctance of rheumatologists to use apps may be lack of evidence [31]. The combination of electronic PROs [28] and objective serology and genetic testing promises to result in the earlier diagnosis of RMDs [32].

Our findings indicate that rheumatologists accept telemedicine to a greater extent than general practitioners. Furthermore, the preferences of rheumatologists differ from those of general practitioners with regard to which specific tools could be implemented into rheumatology care and when. These variations may be related to differences in the two professions as well as the distinct phases of the disease in which rheumatologists and general practitioners encounter RMDs. We also analyzed differences in the acceptance and preferences of telemedicine with regard to the age and gender of the physicians as well as the type and region of their practices. No differences or only small differences were found.

### Perspectives for Telerheumatology

COVID-19 has demonstrated the importance of contactless approaches in medical care. As early as 2018, when we conducted the survey, rheumatologists and general practitioners were willing to use telerheumatology. It is assumed that this readiness has increased due to the pandemic, which is likely to strongly accelerate the use of telemedicine as society adopts new health care standards [18].

Nevertheless, the great potential of telemedicine is not being fully reached. Further research into the implementation of telerheumatology is desperately needed. This includes large-scale randomized controlled studies on the effects and health economic outcomes, as well as risks and adverse events, of specific interventions.

As our results indicate that there will be no “one-size-fits-all” solution in the realm of telemedicine, further research into the perspectives and preferences of physicians, patients, and other telemedicine users in rheumatology is essential. This may provide the foundation for individual patient- and physician-adapted telemedicine options and triage mechanisms to select patients for either digital or analog consultations, as appropriate [25,26].

As physicians reported barriers to the use of telemedicine, it would appear that the structural framework is not yet in place for the effective implementation of telerheumatology. Considerable administrative effort and inadequate reimbursement structures prevented the surveyed physicians from using telemedicine. However, the greatest barrier was the limited knowledge on the part of physicians on the use of telemedicine, which highlights the need for the timely introduction of low-threshold training courses.

### Conclusions

Our study showed that rheumatologists and general practitioners support the implementation of telerheumatology, and two-thirds of respondents would like to implement telemedicine into their clinical routine. Rheumatologists expressed an even greater willingness to use telemedicine than general practitioners, with respondents welcoming a variety of telemedicine approaches. However, only a minority of the surveyed physicians currently use telemedicine. Furthermore, most physicians regard their knowledge of telemedicine as rather poor. The provision of high-quality rheumatology care using telemedicine will require urgently needed research as well as a reduction in existing barriers and training for specialists and generalists.

## Abbreviations

IT: information technology
PRO: patient-reported outcome
RHADAR: RheumaDatenRhePort GbR
RMD: rheumatic and musculoskeletal disease
Telerheumatology: telemedicine in rheumatology
